# Association between daily alcohol consumption and serum alpha klotho levels among U.S. adults over 40 years old: a cross-sectional study

**DOI:** 10.1186/s12889-023-16830-1

**Published:** 2023-10-02

**Authors:** Meihua Jiang, Xiaoyan Tang, Peng Wang, Li Yang, Rui Du

**Affiliations:** 1 Department of Nephrology, General Hospital of Central Theater Command, No. 627, Wuluo Road, Wuhan, Hubei 430070 China; 2 Department of Cardiology, General Hospital of Central Theater Command, No. 627, Wuluo Road, Wuhan, Hubei 430070 China; 3 Department of Radiology, Wuhan Hospital of Traditional Chinese Medicine, No. 303, Sixin Avenue, Wuhan, Hubei 430050 China; 4 Department of Ultrasound, General Hospital of Central Theater Command, No. 627, Wuluo Road, Wuhan, Hubei 430070 China

**Keywords:** Klotho, Alcohol, Aging, NHANES

## Abstract

**Background:**

Klotho is a hormone considered to be an anti-aging biomarker. The relationships between daily alcohol consumption and serum klotho are mainly unknown. The purpose of this study is to assess the relationship between alcohol consumption and serum alpha klotho (α−klotho) levels in the U.S.

**Methods:**

The data came from 11,558 participants aged ≥ 40 in the 2007−2016 National Health and Nutrition Examination Survey. Adults with reliable α−klotho plasma results were the target population. The self-report method was used to assess alcohol consumption. The relationship between daily alcohol intake and serum α−klotho levels was estimated using multivariable linear regression models. We also performed a stratified analysis of clinically important variables.

**Results:**

The mean serum α−klotho level among the 11,558 participants was 843.82 pg/mL. After full adjustment, participants with current moderate and heavy alcohol intake had lower serum α−klotho levels than those who never alcohol intake (*β* =  − 62.64; 95% CI: − 88.86, − 36.43; *P* < 0.001; *β* =  − 81.54; 95% CI: − 111.54, − 51.54; *P* < 0.001, respectively). Furthermore, the stratified analysis indicated that the association was insignificant in individuals with cardiovascular disease, chronic kidney disease, or cancer.

**Conclusion:**

Daily alcohol consumption was inversely associated with serum α−klotho levels among U.S. adults over 40 years old. However, individuals with cardiovascular disease, chronic kidney disease, or cancer found no such relationship.

**Supplementary Information:**

The online version contains supplementary material available at 10.1186/s12889-023-16830-1.

## Introduction

Klotho is a vital aging suppressor protein that has emerged in recent years. The klotho encodes a transmembrane alpha klotho (α−klotho) protein that is abundant in the kidney and brain [1]. After shedding the amino-terminal extracellular domain [2], soluble α-klotho can be found in circulation and circulate in the blood, urine, and cerebrospinal fluid [3]. α−klotho has been reported to be a hormone that exerts different health-related physiological functions, including improvement of insulin sensitivity and glucose uptake [4], modulation of endothelial nitric oxide synthesis to maintain endothelial integrity [5], decreased oxidative stress and chronic inflammation [6]. α−klotho has a preventive effect against systemic disorders and is implicated in the pathophysiology of various diseases, including cardiovascular disease (CVD) [7], chronic kidney disease (CKD) [8], diabetes [9], systemic lupus erythematosus [10], and some kinds of cancer [11].

Alcohol intake significantly impacts life expectancy and contributes considerably to the global burden of morbidity and mortality [12]. It is expected to be the third largest modifiable risk factor for death and disability globally [13]. However, the relationship between alcohol intake and health is complicated. Drinking alcohol has been linked to benefits and harms, because the beneficial effects on certain diseases may offset some problems on others. The amount, type, and pattern of alcohol intake can all affect health outcomes.

Alcohol metabolism produces aldosterone [14], reactive oxygen species [15], and proinflammatory cytokines [16], which contribute to tissue inflammation and damage. α−klotho might exert several functions in alcohol consumption. Several studies have reported the relationship between alcoholic drinks consumption and α−klotho level, but the findings have been conflicting [17–19]. Moreover, previous studies were based on healthy people and small sample sizes. We included participants aged ≥ 40 years from five cycles of a nationally representative sample of U.S. adults in this study to classify alcohol intake in detail and to analyze the relationship between different levels of alcohol consumption and serum α−klotho levels. Furthermore, we investigated whether the relationships differed according to age, sex, BMI, and other variables of interest.

## Methods

### Study design and participants

The National Health and Nutrition Examination Survey (NHANES) is a survey undertaken by the National Center for Health Statistics (NCHS) of the Centers for Disease Control and Prevention in the U.S. NHANES adopts a stratified, multistage, sophisticated probabilistic design to acquire a representative sample of civilians in the U.S. to examine the health and nutritional status of noninstitutionalized citizens. All participant data is a compilation of household interviews, physical examinations in a mobile examination center (MEC), and laboratory testing performed by highly qualified medical experts. More details about NHANES can be found on the website (https://www.cdc.gov/nchs/nhanes/about_nhanes.htm). The NCHS Ethics Review Board authorized the original research, and all participants obtained written informed consent. The current study requires approval by the Institutional Review Board of the author’s institution.

**Fig. 1:**
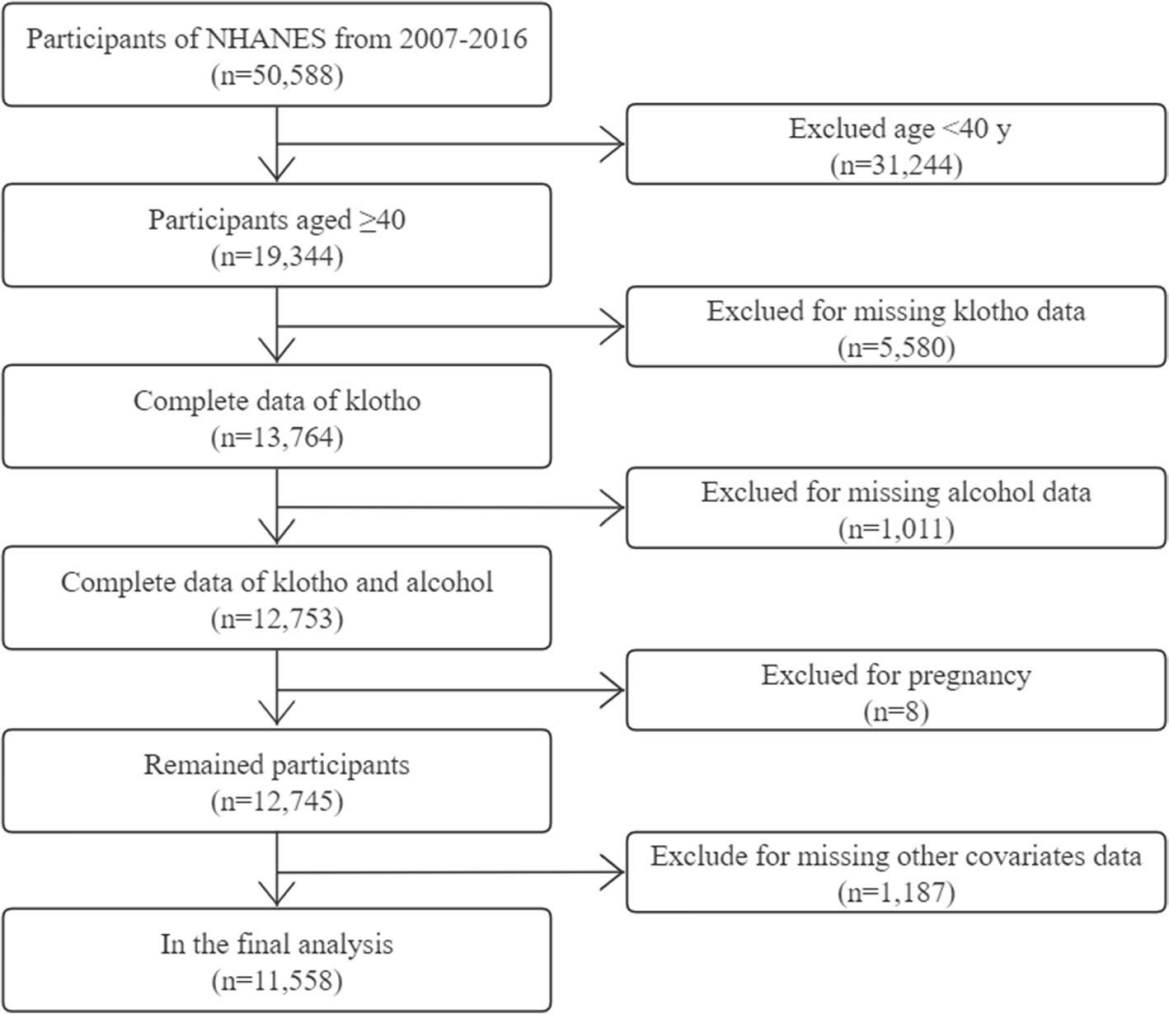
Flowchart of the participants included

We restricted this cross-sectional analysis to participants from the NHANES database from 2007 to 2016 (50,588 individuals in total) because serum α−klotho levels were only tested in NHANES 2007–2016 among participants aged 40–79 who agreed to surplus serum collection for future research. At the time of recruiting, 19,344 were aged 40–79. Of these participants, 5,580 were omitted due to incomplete data on serum α−klotho, missing data on alcohol intake status (*n* = 1,011), pregnancy (*n* = 8), or other covariates (i.e., demographic variables, behavioral factors, history of diseases, *n* = 1,187), and a final analysis sample of 11,558 participants. Figure 1 depicts the participant selection flowchart.

### Alcohol consumption

At the MEC, trained interviewers used the Computer−Assisted Personal Interviewing (CAPI) system method to ask about alcohol intake. Participants were classified into 5 alcohol consumption groups based on the self − reported survey for these questionnaires, as described in previous studies [20, 21]. These are classified as follows: 1) never drinker (had < 12 drinks in a lifetime); 2) former drinker (had ≥ 12 drinks in 1 year and did not drink last year, or did not drink last year but drank ≥ 12 drinks in a lifetime); 3) current light drinker (≤ 1 drink per day for females, ≤ 2 drinks per day for males on average over the past year, or binge drinking [≥ 4 drinks/occasion for females, ≥ 5 drinks/occasion for males] on 1 day per month); 4) current moderate drinker (≤ 2 drinks per day for females, ≤ 3 drinks per day for males, or binge drinking on 2 ~ 5 days per month); 5) current heavy drinker (≥ 3 drinks per day for females, ≥ 4 drinks per day for males, or binge drinking ≥ 5 days per month).

### Klotho level

NHANES Laboratory/Medical Technologists Procedures Manual [22] contains laboratory methodology and protocol information. Serum α−klotho levels in frozen serum samples taken during NHANES 2007–2016 were studied in 2019–2020. Fresh − frozen serum samples, stored at −80^◦^C, were measured using an enzyme-linked immunosorbent assay kit manufactured by IBL International, Japan, per the manufacturer’s instructions. All samples were analyzed twice, and the average value was calculated. The intra-assay precision exhibited a coefficient of variation of 2.3% and 3.3% for two samples. Duplicate tests of the sample yielded 3.8% and 3.4% for the inter-assay coefficients of variation [23]. The mean serum α−klotho level was 698.0 pg/mL, ranging from 285.8 to 1638.6 pg/mL [24].

### Study covariates

Data were collected including race/ethnicity in five categories: Mexican American, other Hispanic, non − Hispanic White, non − Hispanic Black, and Others. Body mass index (BMI) was computed using weight and height (kg/m^2^) and classified into the following World Health Organization (WHO) classes [25]: Normal weight (< 25), Overweight (25−30), and Obese (≥ 30). We collected gamma-glutamyl transferase (GGT) as a marker of oxidative stress, a common disease pathway linking environmental and behavioral exposures to many health outcomes. Education level was classified into 1) Lower than high school, 2) High school or GED, and 3) college and above. Ratio of family income to poverty (PIR) (< 1.30, 1.30–2.99, and ≥ 3.00) [26]. Smoking status was classified into three categories: never smoker (< 100 cigarettes in lifetime); former smoker (≥ 100 cigarettes but not smoke now); and current smoker (≥ 100 cigarettes while smoking now). Diabetes was defined as the presence of any of the following conditions: 1) A self − reported diabetes diagnosis; 2) Anti − diabetic medicine use; 3) Hemoglobin A1c (HbA1c) level of ≥ 6.5% (48 mmol/mol); 4) Fasting plasma glucose level of ≥ 126 mg/dL; 5) Random plasma glucose level of ≥ 200 mg/dL. Hypertension was defined as systolic blood pressure ≥ 140 mmHg and/or diastolic blood pressure ≥ 90 mmHg or being on antihypertensive medication [27]. CKD was defined as eGFR < 60 ml/min/1.73m^2^ and/or urine albumin/creatinine ratio ≥ 30 mg/g according to the Kidney Disease Improving Global Outcomes (KDIGO) 2021 Clinical Practice Guideline for the Management of Glomerular Diseases [28]. Chronic Obstructive Pulmonary Disease (COPD) was defined as having any of the following: 1) FEV1/FVC < 0.7; 2) Ever been informed you had emphysema; 3) Aged over 40, with a history of smoking or chronic bronchitis, and usage of phosphodiesterase − 4 inhibitors, mast cell stabilizers, leukotriene modifiers, or inhaled corticosteroids. The presence of CVD (including congestive heart failure, coronary heart disease, angina pectoris, heart attack, stroke), and cancer depended on whether a doctor had told participants that they had such disease.

### Statistical analysis

Data was acquired from the NHANES project’s nhanesR (http://ckr123.synology.me:3838/nhanesR/). EmpowerStats (version: 4.1. X&Y solutions, Inc., Boston, MA. http://www.empowerstats.com) and R software (version: 4.2.0, The R Foundation; http://www.r-project.org) were used for all analyses. *P* < 0.05 is regarded as statistically significant. Strata, primary sampling units, and sample adult weights were used in studies to account for the National Health Interview Survey’s complicated design. For categorical variables, the weighted Chi − square test was used, while for continuous variables, the weighted linear regression was used. Finally, data were expressed as weighted proportions [95% Confidence interval (CI)] for categorical variables and as weighted means ± Standard Error (SE) for continuous variables. Weighted multiple linear regression models were used to test the relationship between alcohol consumption and serum α−klotho levels. Following the recommendations for Strengthening the Reporting of Observational Studies in Epidemiology (STROBE), tested four models: Crude model had no adjustments. Model 1 minimally adjusted for some demographic factors (age, sex, and race/ethnicity). Model 2 additionally adjusted for lifestyle variables (marital status, education level, PIR, smoking status). Model 3 additionally adjusted for clinical variables (BMI, GGT, diabetes, hypertension, CKD, CVD, COPD, and cancer). Furthermore, stratified analyses were performed to determine whether the relationship between alcohol intake and serum α−klotho levels differed between age groups (< / ≥ 60 years), sex, BMI, hypertension, CKD, CVD, and cancer.

## Results

### Population characteristics

A total of 11,558 participants had a mean age of 56.18 ± 0.16 years, 49.04% males, and 45.35% Non − Hispanic White according to the weighted analysis (Table 1). The mean level of serum α−klotho concentration was 843.82 ± 5.33 pg/ml. Overall, 14.10% of participants were ‘never drinkers’, 21.70% were ‘former drinkers’, 35.11% were ‘current light drinkers’, 13.80% were ‘current moderate drinkers’, and 15.29% were ‘current heavy drinkers’. Each baseline parameter showed statistically significant differences across the 5 alcohol consumption groups (all *P* < 0.001). Serum α−klotho concentration was more likely to fall as alcohol intake rose. Current heavy drinkers were male, more likely to be younger, Non − Hispanic White, smokers, and living alone, had higher GGT, PIR and COPD, lower serum α−klotho levels, and other comorbidities.

**Table 1:** Baseline characteristics of the participants (*N* = 11,558) according to alcohol consumption

**Characteristic**	**Alcohol Consumption Status**					***P***
	**Never Drinker (*N* = 1630)**	**Former Drinker (*N* = 2508)**	**Light Drinker (*N* = 4058)**	**Moderate Drinker (*N* = 1595)**	**Heavy Drinker (*N* = 1767)**	
**Age (years)**	58.65 ± 0.39	58.60 ± 0.30	57.33 ± 0.27	53.99 ± 0.35	51.08 ± 0.25	< 0.001
**α-klotho (pg/mL)**	884.30 ± 11.48	848.21 ± 7.51	854.31 ± 6.90	826.77 ± 9.67	803.14 ± 9.35	< 0.001
**GGT (U/L)**	24.98 ± 0.66	30.10 ± 0.88	26.45 ± 0.46	30.57 ± 2.15	41.03 ± 1.65	< 0.001
**Sex (%)**						< 0.001
Female	72.57 (68.75, 76.08)	50.07 (47.43, 52.71)	45.89 (44.04, 47.76)	65.88 (62.88, 68.76)	40.47 (37.16, 43.87)	
Male	27.43 (23.92, 31.25)	49.93 (47.29, 52.57)	54.11 (52.24, 55.96)	34.12 (31.24, 37.12)	59.53 (56.13, 62.84)	
**Race/ethnicity (%)**						< 0.001
Mexican American	10.52 (7.70, 14.22)	6.87 (5.08, 9.23)	3.32 (2.52, 4.36)	5.50 (4.25, 7.09)	10.74 (8.70, 13.20)	
Other Hispanic	6.71 (5.24, 8.56)	4.61 (3.61, 5.85)	3.10 (2.29, 4.17)	3.55 (2.42, 5.17)	5.91 (4.56, 7.63)	
Non-Hispanic White	56.48 (50.39, 62.38)	71.68 (67.36, 75.64)	80.26 (77.51, 82.75)	89.99 (76.09, 83.40)	70.86 (67.04, 74.41)	
Non-Hispanic Black	12.96 (10.50, 15.89)	10.84 (8.74, 13.37)	7.32 (6.14, 8.70)	8.25 (6.58, 10.30)	8.24 (6.73, 10.05)	
Others	13.33 (10.60, 16.64)	6.01 (4.65, 7.72)	6.00 (4.97, 7.24)	2.70 (1.82, 3.99)	4.25 (3.11, 5.77)	
**BMI (%)**						< 0.001
Normal weight	22.43 (19.85, 25.24)	18.33 (16.02, 20.89)	25.60 (23.53, 27.79)	28.28 (25.25, 31.53)	24.90 (22.18, 27.83)	
Overweight	33.33 (30.33, 36.47)	31.68 (28.97, 34.52)	37.54 (35.51, 39.62)	34.18 (31.06, 37.45)	34.77 (31.60, 38.08)	
Obese	44.24 (41.07, 47.46)	49.99 (47.02, 52.96)	36.86 (34.53, 39.26)	37.53 (34.05, 41.15)	40.33 (36.68, 44.09)	
**Marital status (%)**						< 0.001
Married/living with partner	66.48 (63.07, 69.73)	66.75 (64.34, 69.07)	76.09 (74.24, 77.85)	71.22 (68.01, 74.24)	61.52 (57.89, 65.02)	
Living alone	33.52 (30.27, 36.93)	33.25 (30.93, 35.66)	23.91 (22.15, 25.76)	28.78 (25.76, 31.99)	38.48 (34.98, 42.11)	
**PIR (%)**						< 0.001
< 1.30	28.45 (25.23, 31.91)	25.78 (22.99, 28.8)	10.39 (9.05, 11.90)	11.16 (9.19, 13.48)	23.69 (20.88, 26.76)	
1.30−2.99	33.33 (30.21, 36.61)	33.02 (30.13, 36.04)	21.69 (19.71, 23.82)	23.00 (20.49, 25.72)	28.43 (25.14, 31.97)	
≥ 3.00	38.21 (34.14, 42.46)	41.20 (37.47, 45.04)	67.92 (65.11, 70.61)	65.84 (62.36, 69.16)	47.88 (43.78, 52.00)	
**Education level (%)**						< 0.001
Less than high school	26.87 (23.59, 30.41)	25.18 (22.37, 28.21)	8.96 (7.62, 10.50)	9.54 (8.01, 11.34)	20.59 (17.97, 23.49)	
High school or GED	26.17 (23.01, 29.60)	27.51 (24.84, 30.35)	17.37 (15.68, 19.20)	19.58 (16.66, 22.86)	29.23 (26.02, 32.66)	
Above high school	46.96 (42.30, 51.68)	47.31 (43.48, 51.17)	73.68 (70.97, 76.22)	70.88 (66.94, 74.53)	50.18 (46.46, 53.91)	
**Smoking status (%)**						< 0.001
Never smoker	85.94 (83.71, 87.91)	39.28 (36.45, 42.19)	57.04 (55.01, 59.05)	45.89 (42.64, 49.18)	32.67 (29.77, 35.72)	
Former smoker	9.04 (7.41, 10.98)	38.91 (36.49, 41.39)	31.28 (29.12, 33.52)	35.67 (32.40, 39.09)	26.92 (24.02, 30.03)	
Current smoker	5.02 (3.96, 6.36)	21.81 (19.56, 24.24)	11.68 (10.38, 13.13)	18.43 (16.03, 21.11)	40.40 (37.22, 43.67)	
**Diabetes (%)**						< 0.001
No	71.91 (68.94, 74.70)	71.43 (68.94, 73.80)	82.34 (80.32, 84.20)	87.39 (85.48, 89.08)	85.13 (82.16, 87.67)	
Yes	28.09 (25.30, 31.06)	28.57 (26.20, 31.06)	17.66 (15.80, 19.68)	12.61 (10.92, 14.52)	14.87 (12.33, 17.84)	
**Hypertension (%)**						< 0.001
No	46.07 (42.72, 49.46)	44.39 (41.52, 47.29)	53.31 (50.68, 55.91)	53.88 (50.41, 57.32)	54.31 (51.20, 57.39)	
Yes	53.93 (50.54, 57.28)	55.61 (52.71, 58.48)	46.69 (44.09, 49.32)	46.12 (42.68, 49.59)	45.69 (42.61, 48.80)	
**CKD (%)**						< 0.001
No	79.15 (76.11, 81.90)	76.83 (74.63, 78.90)	85.67 (84.24, 86.99)	86.47 (83.81, 88.75)	86.34 (84.48, 88.00)	
Yes	20.85 (18.10, 23.89)	23.17 (21.10, 25.37)	14.33 (13.01, 15.76)	13.53 (11.25, 16.19)	13.66 (12.00, 15.52)	
**CVD (%)**						< 0.001
No	89.13 (87.10, 90.87)	80.23 (77.80, 82.45)	89.96 (88.96, 90.89)	92.95 (90.93, 94.55)	91.95 (90.08, 93.49)	
Yes	10.87 (9.13, 12.90)	19.77 (17.55, 22.20)	10.04 (9.11, 11.04)	7.05 (5.45, 9.07)	8.05 (6.51, 9.92)	
**COPD (%)**						< 0.001
No	95.49 (93.69, 96.79)	88.27 (86.40, 89.91)	93.10 (91.95, 94.10)	92.98 (91.12, 94.47)	92.84 (90.69, 94.52)	
Yes	4.51 (3.21, 6.31)	11.73 (10.09, 13.60)	6.90 (5.90, 8.05)	7.02 (5.53, 8.88)	7.16 (5.48, 9.31)	
**Cancer (%)**						< 0.001
No	88.01 (85.32, 90.27)	85.03 (83.21, 86.68)	84.20 (82.67, 85.63)	86.42 (83.94, 88.56)	91.00 (88.93, 92.71)	
Yes	11.99 (9.73, 14.68)	14.97 (13.32, 16.79)	15.80 (14.37, 17.33)	13.58 (11.44, 16.06)	9.00 (7.29, 11.07)	

Mean ± SE for continuous variables: *P* value for weighted linear regression model. % (95% Confidence interval CI) for categorical variables: *P* value for weighted chi-square test

*Abbreviations*: *GGT* Gamma-glutamyl transferase, *BMI* Body mass index, *PIR* Ratio of family income to poverty, *CKD* Chronic kidney disease, *CVD* Cardiovascular disease, *COPD* Chronic obstructive pulmonary disease

Univariate examination of the potential factors affecting the serum α−klotho level, as given in Supplementary Table S1, revealed that as alcohol consumption rose, the concentration of α−klotho level in the serum dropped (*P* < 0.001). Furthermore, age, sex, race/ethnicity, BMI, smoking status, hypertension, CKD, CVD, and cancer were linked to serum α−klotho levels.

### Relationship between alcohol consumption and serum α−klotho level

Four multivariate linear regression models were conducted to assess the association between daily alcohol intake and serum α−klotho level, as shown in Table 2. In the crude model, no factors were modified, and the serum α−klotho level at current moderate and heavy alcohol consumption groups were substantially lower (*β* =  − 57.53; 95% CI: − 82.69, − 32.36;* P* < 0.001; *β* =  − 81.16; 95% CI: − 109.13, − 53.19; *P* < 0.001, respectively) than never alcohol consumption group. After adjusting for age, sex, and race/ethnicity, the correlation with serum α−klotho level remained significantly negative in model 1 (*β* =  − 63.65; 95% CI: − 90.31, − 36.99; *P* < 0.001; *β* =  − 88.41; 95% CI: − 116.74, − 60.07; *P* < 0.001, respectively). After additionally adjusted for marital status, education level, PIR, and smoking status, the correlation with serum α−klotho level remained significantly negative in model 2 (*β* =  − 60.34; 95% CI: − 86.59, − 34.09; *P* < 0.001; *β* =  − 78.52; 95% CI: − 108.10, − 48.94; *P* < 0.001, respectively). The result in fully adjusted model 3 is consistent with the prior finding (*β* =  − 62.64; 95% CI: − 88.86, − 36.43; *P* < 0.001; *β* =  − 81.54; 95% CI: − 111.54, − 51.54; *P* < 0.001, respectively). Furthermore, compared to the never alcohol consumption group, the serum α−klotho level in the former and current light alcohol consumption groups was significantly lower in the crude model (*β* =  − 36.09; 95% CI: − 62.27, − 9.91; *P* = 0.008; *β* =  − 29.99; 95% CI: − 53.51, − 6.46; *P* = 0.015, respectively). In model 1, serum α−klotho level in the former alcohol consumption group were significantly lower (*β* =  − 26.17; 95% CI: − 51.51, − 0.84; *P* = 0.047), while there was no statistical difference in the level of serum α−klotho level between the current light alcohol consumption group and never alcohol consumption group (*β* =  − 18.91; 95% CI: − 42.30, 4.48; *P* = 0.118). The relationship was not significant in both former and current light alcohol intake groups in model 2 (*β* =  − 19.00; 95% CI: − 45.94, 7.94; *P* = 0.172; *β* =  − 18.43; 95% CI: − 41.80, 4.94; *P* = 0.127, respectively) and model 3 (*β* =  − 17.02; 95% CI: − 44.52, 10.47; *P* = 0.220; *β* =  − 20.06; 95% CI: − 43.71, 3.59; *P* = 0.095, respectively).

**Table 2:** Association between alcohol consumption and serum α−klotho level

**Alcohol consumption**	Crude model		Model 1		Model 2		Model 3	
	**β (95% CI)**	***P***	**β (95% CI)**	***P***	**β (95% CI)**	***P***	**β (95% CI)**	***P***
Never drinker	Ref		Ref		Ref		Ref	
Former drinker	−36.09 (−62.27 ~ −9.91)	0.008	−26.17 (−51.51 ~ −0.84)	0.047	−19.00 (−45.94 ~ 7.94)	0.172	−17.02 (−44.52 ~ 10.47)	0.220
Light drinker	−29.99 (−53.51 ~ −6.46)	0.015	−18.91 (−42.30 ~ 4.48)	0.118	−18.43 (−41.80 ~ 4.94)	0.127	−20.06 (−43.71 ~ 3.59)	0.095
Moderate drinker	−57.53 (−82.69 ~ −32.36)	< 0.001	−63.65 (−90.31 ~ −36.99)	< 0.001	−60.34 (−86.59 ~ −34.09)	< 0.001	−62.64 (−88.86 ~ −36.43)	< 0.001
Heavy drinker	−81.16 (−109.13 ~ −53.19)	< 0.001	−88.41 (−116.74 ~ −60.07)	< 0.001	−78.52 (−108.10 ~ −48.94)	< 0.001	−81.54 (−111.54 ~ −51.54)	< 0.001
*P* for trend		< 0.001		< 0.001		< 0.001		< 0.001

β was the effect size (pg/mL) of the change in serum α−klotho level, and the 95%CI indicated the 95% Confidence interval

Crude model adjusted for: none

Model 1 adjusted for: age, sex, race/ethnicity

Model 2 adjusted for: age, sex, race/ethnicity, marital status, education level, PIR, smoking status

Model 3 adjusted for: age, sex, race/ethnicity, BMI, GGT, marital status, education level, PIR, smoking status, diabetes, hypertension, CKD, CVD, COPD, cancer

*Abbreviations*: *PIR* Ratio of family income to poverty, *BMI* Body mass index, *GGT* Gamma-glutamyl transferase, *CKD* Chronic kidney disease, *CVD* Cardiovascular disease, *COPD* Chronic obstructive pulmonary disease

The correlation between alcohol consumption and serum α−klotho levels was consistent across subgroups stratified by age group, sex, BMI, hypertension, and cancer in the stratified analyses (Table 3). The connection, however, was not significant in individuals with CKD, CVD, or cancer.

**Table 3:** Stratified analyses of association between alcohol consumption and serum α−klotho levels concentrations (pg/mL)

	**Alcohol consumption**					***P* for interaction**
	**Never**	**Former**	**Current Light**	**Current Moderate**	**Current Heavy**	
**Age**						0.970
40−59	0	−16.96(−57.20 ~ 23.29)	−24.15(−58.56 ~ 10.25)	−63.46(−96.82 ~ −27.09)	−80.85(−118.68 ~ −43.01)	
60−79	0	−17.16(−48.80 ~ 14.48)	−12.23(−41.28 ~ 16.80)	−58.17(−92.59 ~ −23.75)	−80.88(−122.34 ~ −39.41)	
**Sex**						0.240
Female	0	−31.07(−63.78 ~ 1.65)	−26.99(−54.34 ~ 0.36)	−84.23(−111.99 ~ −56.47)	−111.15(−156.42 ~ −65.88)	
Male	0	5.17(−52.31 ~ 62.64)	−4.18(− 49.43 ~ 41.07)	−34.49(−85.92 ~ 16.94)	−56.77(−106.37 ~ −7.18)	
**BMI**						0.196
Normal weight	0	−30.14(−78.33 ~ 18.05)	−10.13(−58.34 ~ 38.09)	−42.51(−91.59 ~ 6.57)	−71.06(−131.76 ~ −10.36)	
Overweight	0	−7.97(−49.48 ~ 33.53)	−39.45(−75.68 ~ −3.22)	−73.45(−124.93 ~ −21.98)	−91.73(−150.94 ~ −32.51)	
Obese	0	−17.15(−58.63 ~ 24.33)	−7.10(−42.51 ~ 28.30)	−68.33(−109.06 ~ −27.59)	−80.30(−120.209 ~ −40.52)	
**Hypertension**						0.986
No	0	−1.84(−35.35 ~ 31.67)	−5.45(−39.01 ~ 28.10)	−48.65(−82.41 ~ −14.89)	−63.34(−100.64 ~ −26.04)	
Yes	0	−27.88(−68.45 ~ 12.69)	−32.18(−67.49 ~ 3.12)	−74.70(−115.86 ~ −33.53)	−99.83(−142.09 ~ −57.56)	
**CKD**						0.088
No	0	−20.88(−50.38 ~ 8.63)	−25.79(−50.06 ~ 0.47)	−66.34(−95.78 ~ −36.91)	−93.29(−126.79 ~ −59.80)	
Yes	0	5.90(−55.30 ~ 67.09)	3.56(−47.97 ~ 55.08)	−50.84(−127.78 ~ 26.11)	−14.91(−84.12 ~ 54.29)	
**CVD**						0.528
No	0	−13.14(−43.39 ~ 17.12)	−20.66(−45.86 ~ 4.55)	−64.79(−92.74 ~ −36.85)	−83.92(−114.63 ~ −53.21)	
Yes	0	−25.86(−85.07 ~ 33.34)	−10.29(−77.68 ~ 57.09)	−24.43(−94.22 ~ 45.36)	−49.38(−142.61 ~ 43.86)	
**Cancer**						0.156
No	0	−15.93(−44.80 ~ 12.93)	−22.32(−49.64 ~ 1.01)	−59.13(−87.15 ~ −31.11)	−84.12(−116.08 ~ −52.16)	
Yes	0	−19.40(−89.77 ~ 50.96)	8.24(−54.95 ~ 71.45)	−82.96(−160.92 ~ −5.01)	−51.04(−126.00 ~ 23.93)	

Each stratification adjusted for all factors (age, sex, race/ethnicity, BMI, GGT, marital status, education level, PIR, smoking status, diabetes, hypertension, CKD, CVD, COPD, and cancer) except the stratification factor itself

*Abbreviations*: *BMI* Body mass index, *GGT* Gamma-glutamyl transferase, *PIR* Ratio of family income to poverty, *CKD* Chronic kidney disease, *CVD* Cardiovascular disease, *COPD* Chronic obstructive pulmonary disease

## Discussion

This analysis of nationally representative data found that alcohol intake had different relationships with serum α−klotho levels among U.S. adults over 40 years old. Current moderate and heavy drinking was inversely associated with serum α−klotho levels. However, this association was not significant in individuals with CKD, CVD, or cancer. Serum α−klotho levels were not significantly associated with former or current light alcohol drinking. These results provide evidence for the relationship between drinking and aging.

There is no link between current drinking and mortality, although high intake has been linked to an increased risk of mortality [29, 30]. Previously, alcohol use was a major contributor to declining life expectancy [31]. This study investigates the negative relationship between moderate and heavy drinking in a potent aging biomarker known as serum α−klotho level. Our findings disagreed with several earlier studies [18, 19] but agreed with a recent study [17]. In a recent cross-sectional investigation of 139 sedentary, healthy people aged 18 to 25 years, increased alcohol intake was found to be directly related to higher S-klotho levels in women [18]. Some investigations found that non-cirrhotic alcoholics had much lower klotho levels than controls but significantly higher in liver cirrhosis [32, 33]. Another study of 74 sedentary healthy adults aged 40 to 65 found that consuming total alcoholic drinks was related to decreased S-klotho plasma levels [17]. However, in these studies, the sample size was small.

The present study looked at people who consumed alcohol, both past and now drinking, including heavy drinkers. We extended previous findings by demonstrating that alcohol consumption was still inversely related to serum α−klotho levels. The particular processes underpinning the synergy between alcohol consumption and α−klotho remain unknown. It may be related to the following mechanisms: 1. Alcohol intake can increase the aldosterone levels [14], and aldosterone lowers renal levels of αKlotho mRNA and protein [34]. 2. Reactive oxygen species are a byproduct of alcohol metabolism and contribute to oxidative stress [15]. It has been demonstrated that oxidative stress reduces the expression of the α-klotho gene [35]. 3. Alcohol use enhances the secretion of proinflammatory cytokines such as tumor necrosis factor (TNF), interferon γ (IFN- γ), and interleukin-6 (IL-6) [16], downregulates α−klotho gene expression [36]. 4. Chronic alcohol intake has been demonstrated to activate and inhibit autophagy [37]. The α−klotho has been identified as a possible autophagy regulator [38]. All the previous mechanisms may suggest that alcohol intake could be important in the α−klotho levels.

Furthermore, our study showed that the correlation between alcohol consumption and serum α−klotho levels appeared more robust in individuals without CKD compared to those with CKD. α−klotho is primarily produced in the distal convoluted tubules, and lower levels of α−klotho have been linked to a decline in kidney function [39]. Serum levels of α−klotho decreased as CKD progresses, even as early as stage 2 CKD [40]. The progression of CKD leads to increased fibroblast growth factor 23 expression and decreased active vitamin D levels, which ultimately suppress α-klotho expression [6]. Our study suggests that alcohol may have a more pronounced effect on reducing α-klotho concentration in individuals without kidney disease. In our study, alcohol consumption was not significantly associated with serum α−klotho levels among the participants with CVD or cancer. It could be because CVD and cancer are more directly related to serum α−klotho levels [11, 41].

This study has some significance as it sheds light on the need to control alcohol consumption concerning aging. Our findings highlight a potential public health concern, the findings offer supportive evidence for clinical work. The results of our study align with the Dietary Guidelines for Americans, 2020–2025 [42], which recommend limiting alcohol intake to a maximum of 2 drinks per day for men and 1 drink per day for women. All adults must restrict their alcohol consumption. Our research findings can serve as a valuable reference.

### Strengths and limitations

This study’s strengths included a large sample size, utilizing a representative, multiracial population, and improved generalizability to the U.S. population. Nonetheless, the current study has several limitations. First, a bias may exist for the self − reporting alcohol consumption status may be a discrepancy with the actual drinking status in the database, such erroneous reports may generate a bias toward the null hypothesis for the result. Second, our study is an observational and cross-sectional design, limiting our ability to establish causal relations. Third, we can’t acquire an accurate dose of the alcohol consumption in the NHANES database, which may prevent a further study between alcohol consumption and α−klotho levels. Fourth, although we considered a variety of confounding factors, we cannot exclude the possibility of residual confounding, such as dietary intake, physical activity, and liver function.

## Conclusions

In summary, current moderate and heavy alcohol consumption was inversely associated with serum α−klotho levels in middle-aged and older adults. It suggested that alcohol drinking may induce aging by regulating α-klotho levels. However, this relationship was insignificant in individuals with CKD, CVD, or cancer. More research is warranted to explore these interactions further.

### Supplementary Information

**Additional file 1: Table S1.** Univariate analysis for serum α-klotho level (pg/ml).

## Data Availability

Details about NHANES can be found on the website (https://www.cdc.gov/nchs/nhanes/about_nhanes.htm). All data generated or analyzed during this study are included in this article and its supplementary material files. Further enquiries can be directed to the corresponding author.

## Abbreviations

NHANES: National Health and Nutrition Examination Survey
NCHS: National Center for Health Statistics
MEC: Mobile examination center
BMI: Body mass index
GGT: Gamma-glutamyl transferase
CI: Confidence interval
CKD: Chronic kidney disease
CVD: Cardiovascular disease
COPD: Chronic Obstructive Pulmonary Disease
WHO: World Health Organization
KDIGO: Kidney Disease Improving Global Outcomes
