# Supplementary material for: Association between daily alcohol consumption and serum alpha klotho levels among U.S. adults over 40 years old: a cross-sectional study
Source: BMC Public Health. 2023 Oct 2;23:1901. doi: 10.1186/s12889-023-16830-1 (PMC10544600; doi:10.1186/s12889-023-16830-1)
Supplement: Supplementary file 1 — Additional file 1: Table S1. Univariate analysis for serum α-klotho level (pg/ml). [file 12889_2023_16830_MOESM1_ESM.docx]

**Table S1** Univariate analysis for serum α-klotho level (pg/ml)

|  | **N** | **Serum α-klotho level**  **Mean (95%CI)** | **β (95% CI)** | ***P*** |
| --- | --- | --- | --- | --- |
| **Age** | 11558 | 843.82 (833.37, 854.28) | -2.20 (-2.83, -1.58) | <0.001 |
| **Sex** |  |  |  |  |
| Female | 5890 | 861.05 (848.15, 873.95) | Ref. |  |
| Male | 5668 | 825.31 (813.00, 837.61) | -35.74 (-49.81, -21.68) | <0.001 |
| **Race/ethnicity** |  |  |  |  |
| Mexican American | 1768 | 852.68 (833.89, 871.47) | Ref. |  |
| Other Hispanic | 1247 | 873.51 (848.40, 898.62) | 20.83 (-8.59, 50.25) | 0.169 |
| Non-Hispanic White | 5241 | 833.70 (822.26, 845.14) | -18.98 (-39.93, 1.96) | 0.080 |
| Non-Hispanic Black | 2282 | 907.55 (882.56, 932.54) | 54.87 (24.43, 85.31) | <0.001 |
| Others | 1020 | 846.30 (821.06, 871.53) | -6.39 (-34.12, 21.35) | 0.653 |
| **BMI** |  |  |  |  |
| Normal weight | 2696 | 867.96 (849.25, 886.66) | Ref. |  |
| Overweight | 4001 | 836.45 (820.51, 852.40) | -31.51 (-54.04, -8.98) | 0.008 |
| Obese | 4861 | 835.74 (824.29, 847.19) | -32.22 (-51.82, -12.61) | 0.002 |
| **Marital status** |  |  |  |  |
| Married/living with partner | 7474 | 839.48 (827.74, 851.21) | Ref. |  |
| Living alone | 4084 | 854.16 (839.81, 868.50) | 14.68 (-0.78, 30.14) | 0.067 |
| **PIR** |  |  |  |  |
| <1.30 | 3458 | 846.66 (832.49, 860.83) | Ref. |  |
| 1.30−2.99 | 3511 | 840.41 (824.35, 856.47) | -6.25 (-24.68, 12.18) | 0.508 |
| ≥3.00 | 4589 | 844.53 (831.47, 857.60) | -2.13 (-19.37, 15.12) | 0.810 |
| **Education level** |  |  |  |  |
| Less than high school | 3068 | 844.57 (825.97, 863.18) | Ref. |  |
| High school or GED | 2584 | 822.66 (807.99, 837.32) | -21.92 (-45.15, 1.31) | 0.068 |
| Above high school | 5906 | 851.22 (837.34, 865.09) | 6.64 (-14.47, 27.75) | 0.539 |
| **Alcohol consumption** |  |  |  |  |
| Never drinker | 1630 | 884.30 (861.80, 906.80) | Ref. |  |
| Former drinker | 2508 | 848.21 (833.49, 862.93) | -36.09 (-62.27, -9.91) | 0.009 |
| Light drinker | 4058 | 854.31 (840.79, 867.83) | -29.99 (-53.51, -6.46) | 0.015 |
| Moderate drinker | 1595 | 826.77 (807.82, 845.72) | -57.53 (-82.69, -32.36) | <0.001 |
| Heavy drinker | 1767 | 803.14 (784.81, 821.46) | -81.16 (-109.13, -53.19) | <0.001 |
| **Smoking status** |  |  |  |  |
| Never smoker | 5831 | 862.37 (849.09, 875.65) | Ref. |  |
| Former smoker | 3451 | 827.67 (813.42, 841.92) | -34.70 (-49.96, -19.43) | <0.001 |
| Current smoker | 2276 | 818.90 (801.38, 836.42) | -43.47 (-62.75, -24.18) | <0.001 |
| **GGT** | 11558 | 843.82 (833.37, 854.28) | 0.21 (-0.09, 0.05) | 0.169 |
| **Diabetes** |  |  |  |  |
| No | 8588 | 843.38 (832.22, 854.54) | Ref. |  |
| Yes | 2970 | 845.67 (828.93, 862.41) | 2.29 (-14.79, 19.38) | 0.794 |
| **Hypertension** |  |  |  |  |
| No | 5265 | 853.82 (842.27, 865.37) | Ref. |  |
| Yes | 6293 | 833.32 (820.57, 846.07) | -20.50 (-32.85, -8.16) | 0.002 |
| **CKD** |  |  |  |  |
| No | 9200 | 851.87 (840.37, 863.37) | Ref. |  |
| Yes | 2358 | 802.60 (786.71, 818.49) | -49.27 (-66.94, -31.60) | <0.001 |
| **CVD** |  |  |  |  |
| No | 9963 | 848.14 (837.32, 858.97) | Ref. |  |
| Yes | 1595 | 809.11 (789.62, 828.59) | -39.03 (-58.25, -19.82) | <0.001 |
| **COPD** |  |  |  |  |
| No | 10682 | 845.11 (834.29, 855.93) | Ref. |  |
| Yes | 876 | 828.10 (802.21, 853.98) | -17.01 (-43.70, 9.67) | 0.215 |
| **Cancer** |  |  |  |  |
| No | 10159 | 848.60 (838.14, 859.05) | Ref. |  |
| Yes | 1399 | 814.13 (795.75, 832.51) | -34.47 (-51.01, -17.93) | <0.001 |

Note. Mean (95%CI): survey-weighted mean (95% CI) for Serum α-klotho level.

**Abbreviations:** BMI, body mass index; PIR, Ratio of family income to poverty; GGT, Gamma-glutamyl transferase; CKD, chronic kidney disease; CVD, cardiovascular disease; COPD, chronic obstructive pulmonary disease; 95%CI: 95% Confidence interval.
